# IgG4-Related Lymphadenopathy

**DOI:** 10.1155/2012/572539

**Published:** 2012-06-10

**Authors:** Yasuharu Sato, Tadashi Yoshino

**Affiliations:** Department of Pathology, Okayama University Graduate School of Medicine, Dentistry and Pharmaceutical Sciences, 2-5-1 Shikata-cho, Kita-ku, Okayama 700-8558, Japan

## Abstract

Lymphadenopathy is frequently observed in patients with immunoglobulin G4-related disease (IgG4-RD) and sometimes appears as the first manifestation of the disease. The diagnosis of IgG4-related lymphadenopathy is complicated owing to a great histological diversity, with at least 5 histological subtypes. Indeed, lymph node biopsy may be performed under the suspicion that the lymphadenopathy is a malignant lymphoma or other lymphoproliferative disorder. The diagnosis of IgG4-RD is characterized by both elevated serum IgG4 (>135 mg/dL) and histopathological features, including a dense lymphoplasmacytic infiltrate rich in IgG4^+^ plasma cells (IgG4^+^/IgG^+^ plasma cell ratio >40%). However, patients with hyper-interleukin (IL-) 6 syndromes such as multicentric Castleman's disease, rheumatoid arthritis, and other immune-mediated conditions frequently show lymph node involvement and often fulfill the diagnostic criteria for IgG4-RD. Owing to these factors, IgG4-RD cannot be differentiated from hyper-IL-6 syndromes on the basis of histological findings alone. Laboratory analyses are crucial to differentiate between the 2 diseases. Hyper-IL-6 syndromes are characterized by elevated serum levels of IgG, IgA, IgM, and C-reactive protein (CRP); thrombocytosis; anemia; hypoalbuminemia; hypocholesterolemia. In contrast, IgG4-RD does not share any of these characteristics. Therefore, the diagnosis of IgG4-RD requires not only pathological findings but also clinical and laboratory analyses.

## 1. Introduction

Immunoglobulin G4-related disease (IgG4-RD) frequently involves lymph nodes in a localized or systemic fashion [1–3]. Indeed, approximately 80% of patients with autoimmune pancreatitis (IgG4-related pancreatitis) has lymphadenopathy, most commonly involving the mediastinal and intraabdominal lymph nodes [4]. Moreover, lymphadenopathy sometimes appears as the first manifestation of IgG4-RD [1–3].

IgG4-RD is an inflammatory condition characterized by a dense lymphoplasmacytic infiltrate rich in IgG4^+^ plasma cells; an IgG4^+^/IgG^+^ plasma cell ratio of >40% is an important diagnostic criterion for the disease [3, 5]. Patients with IgG4-related lymphadenopathy occasionally show systemic lymphadenopathy and elevated serum levels of IgG4 and IgE, and less often show low titers of various autoantibodies [1–3, 5, 6]. Therefore, the disease often shares clinical characteristics with malignant lymphoma, multicentric Castleman's disease, and immune-mediated conditions [1–3, 7, 8]. However, the patients often show an excellent response to steroid therapy and do not show the B symptoms of fever, fatigue, weight loss, and night sweats. Moreover, no monoclonal immunoglobulin gene rearrangement is observed [1, 3].

Recently, several studies dealing with the morphological and immunohistological findings of IgG4-related lymphadenopathy have been performed [1–3]. Furthermore, these studies have shown that lymphadenopathies are histologically distinct from the effects of IgG4-RD on other organs (i.e., storiform fibrosis and obliterative phlebitis are usually absent) [1–3]. From this histological diversity, we consider the presence of 5 subtypes of IgG4-related lymphadenopathy (Table 1): multicentric Castleman's disease-like, reactive follicular hyperplasia-like, interfollicular expansion and immunoblastosis, progressively transformed germinal center (PTGC-) type, and inflammatory pseudotumor-like IgG4-related lymphadenopathy [1–3].

## 2. Clinical and Pathological Features of IgG4-Related Lymphadenopathy

### 2.1. Type I: Multicentric Castleman's Disease-Like

**Figure 1:**
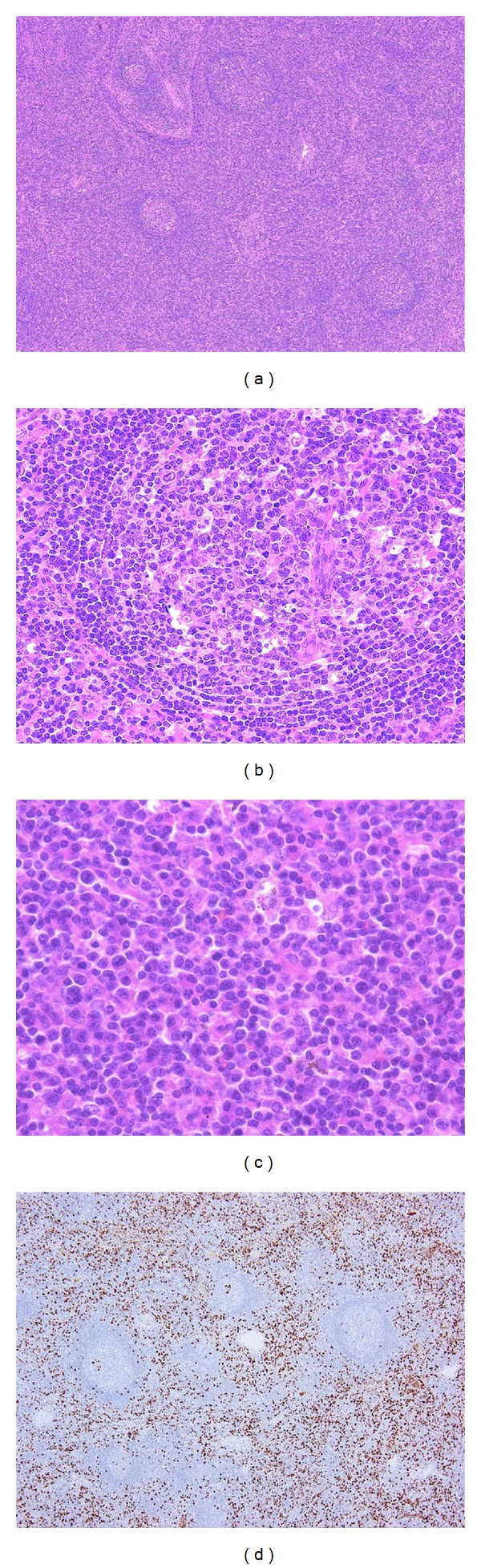
IgG4-related lymphadenopathy (type I). (a) The lymph node shows interfollicular expansion with normal to hyperplastic germinal centers. (b) The germinal centers are penetrated by blood vessels. (c) A large number of mature plasma cells with small lymphocytes are seen. (d) Immunostaining shows numerous IgG4^+^ cells in the interfollicular zone.

This type is frequently characterized by systemic lymphadenopathy [1–3]. Histologically, the lymph node shows interfollicular expansion with normal to hyperplastic germinal centers, penetrated by blood vessels. Abundant plasma cells and scattered eosinophils are apparent in the interfollicular zone (Figure 1). Although these features are similar to the features of multicentric Castleman's disease (MCD), MCD is usually characterized by the presence of small and regressive germinal centers and no eosinophil infiltration [8]. However, pathological diagnosis is difficult, because MCD sometimes fulfills the diagnostic criteria for IgG4-RD, namely, abundant IgG4^+^ plasma cell infiltration (i.e., IgG4^+^/IgG^+^ plasma cell ratio >40%) and elevated serum IgG4 levels [8]. Therefore, the 2 diseases cannot be differentiated on the basis of histological findings alone, and laboratory analyses are critical for a definitive diagnosis (Table 2).

### 2.2. Type II: Reactive Follicular Hyperplasia-Like

**Figure 2:**
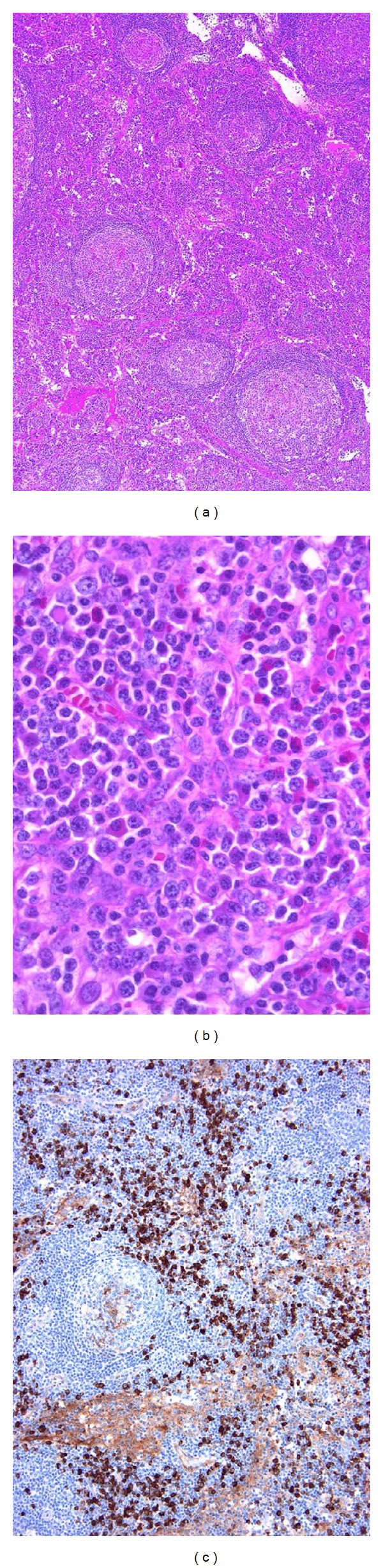
IgG4-related lymphadenopathy (type II). (a) The lymph node shows reactive follicular hyperplasia with intact sinuses. (b) A small to moderate number of mature plasma cells with small lymphocytes and eosinophils are present. (c) Immunostaining shows numerous IgG4^+^ cells in the interfollicular zone.

The lymph nodes usually exhibit reactive follicular hyperplasia, and sinuses are intact. The reactive follicles comprise a germinal center surrounded by a discrete mantle zone. The interfollicular zone contains a small to moderate number of mature plasma cells, with small lymphocytes and eosinophils (Figure 2). This type is frequently found in the regional lymph nodes of IgG4-RD [1, 2].

### 2.3. Type III: Interfollicular Expansion and Immunoblastosis

**Figure 3:**
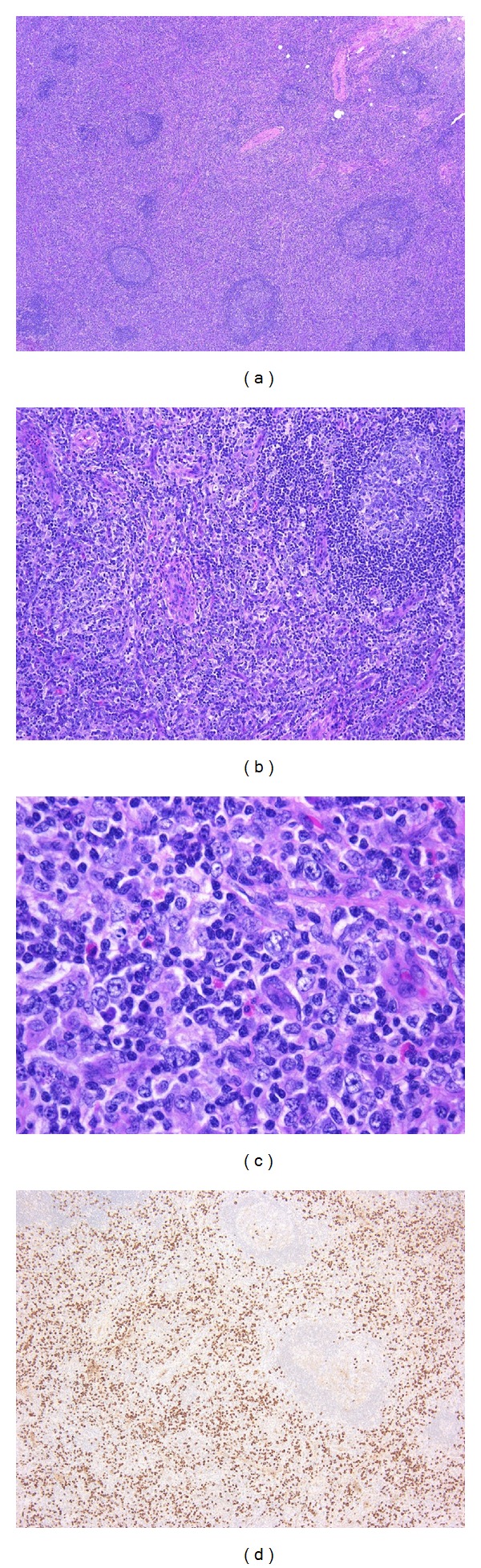
IgG4-related lymphadenopathy (type III). (a) The lymph node shows interfollicular expansion with normal to small germinal centers. (b) Hypervascular proliferation is seen in the interfollicular zone. (c) A mixed infiltrate of small lymphocytes, immunoblasts, immature plasma cells, mature plasma cells, and scattered eosinophils is observed (d) Numerous IgG4^+^ cells are present in the interfollicular zone.

This type is also frequently characterized by systemic lymphadenopathy [1–3]. Histologically, the lymph nodes show marked interfollicular expansion with prominent high endothelial venules and patent sinuses. The lymphoid follicles are usually normal to atrophic. A mixed infiltrate of small lymphocytes, immunoblasts, immature plasma cells, mature plasma cells, and scattered eosinophils is observed (Figure 3). The morphological features overlap with those of atypical lymphoplasmacytic and immunoblastic proliferation (ALPIBP), which is a characteristic lymphadenopathy observed in patients with rheumatoid arthritis (RA), systemic lupus erythematosus (SLE), and other autoimmune diseases [9].

This type is somewhat similar to angioimmunoblastic T-cell lymphoma. However, it is noteworthy that these lesions lack clusters of clear cells and definite cytologic atypia typical of the lymphoma. Moreover, CD21^+^ follicular dendritic cell proliferation, the presence of CD10^+^ T-cells, and T-cell receptor gene rearrangement are not observed [1–3].

### 2.4. Type IV: Progressively Transformed Germinal Centers (PTGC)-Type

**Figure 4:**
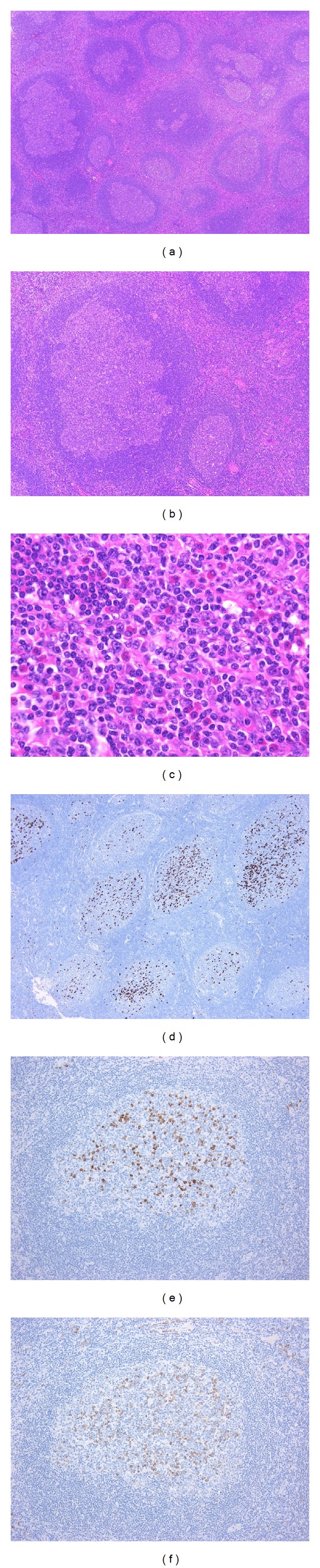
IgG4-related lymphadenopathy (type IV). (a) The lymph node shows marked follicular hyperplasia with PTGC. (b) The PTGCs appear as round to oval structures 2-3 times the diameter of the other reactive follicles. (c) Numerous eosinophils infiltrate the interfollicular zone. (d) The majority of IgG4^+^ plasma cells reside in the germinal centers, with a small number present in the interfollicular zone. (e), (f): The IgG4^+^/IgG^+^ plasma cell ratio is >40% (e: IgG4-immunostain, f: IgG-immunostain).

PTGC is a benign condition of unknown origin characterized by reactive follicular hyperplasia in the lymph nodes [10, 11]. Recently, we were the first to report cases of patients with IgG4-RD in PTGC of lymph nodes (PTGC-type IgG4-related lymphadenopathy) [3]. In this type, the lymph nodes demonstrate numerous lymphoid follicles with hyperplastic germinal centers and a distinct mantle zone but no expansion of the interfollicular zone. PTGCs are also apparent, appearing as round to oval structures with diameters 2 or 3 times the diameter of the other reactive follicles. They are predominantly composed of small lymphocytes, centrocytes, centroblasts, and numerous mature plasma cells and plasmacytoid cells. The interfollicular zone shows infiltration by numerous eosinophils, whereas T zones are indistinct (Figure 4). Interestingly, a unique feature of this type is the localization of the majority of IgG4^+^ plasma cells in the germinal centers, with only a small number present in the interfollicular zone [12]. However, in a few cases of this type, IgG4^+^ plasma cells are detected in both the germinal centers and interfollicular zone [12].

 Patients with this type have a uniform clinicopathology. The patients initially present with asymptomatic localized submandibular lymphadenopathy, with half of them showing progression to extranodal IgG4-RD, systemic disease, or both during the follow-up period [12].

### 2.5. Type V: Inflammatory Pseudotumor (IPT)-Like

**Figure 5:**
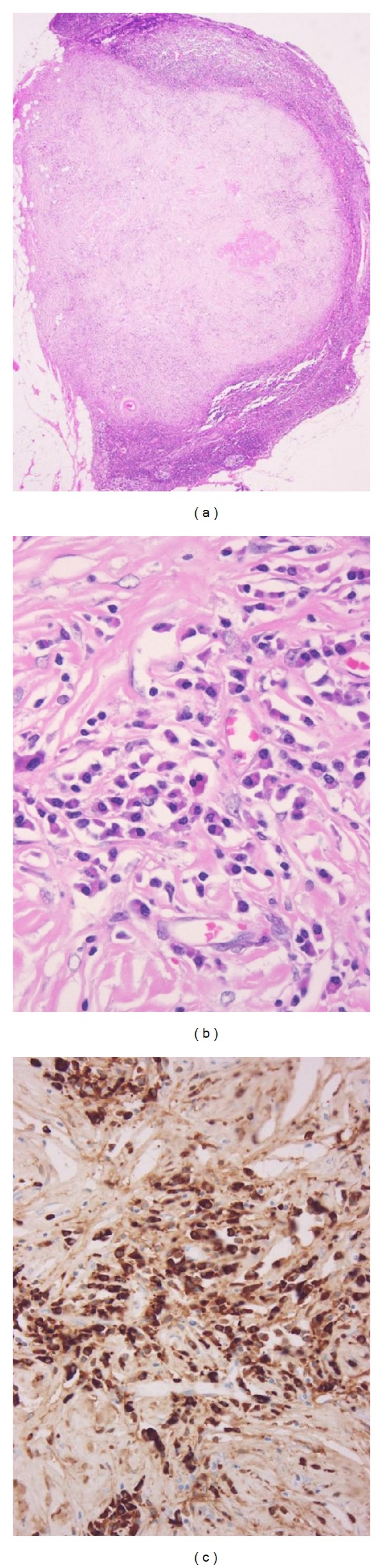
IgG4-related lymphadenopathy (type V). This is a regional lymph node with IgG4-related cholangitis. (a) The majority of the lymph node is replaced by hyalinized fibrous tissue. (b) Mature plasma cells infiltrate the hyalinized fibrous tissue. (c) The mature plasma cells are IgG4^+^.

In this type, the lymph nodes show asymptomatic localized lymphadenopathy [13]. Histologically, most of the lymph node is occupied by hyalinized fibrous tissue. A few residual lymphoid follicles with hyperplastic germinal centers and a focally dense lymphoid infiltrate are observed in the lymph node. Small lymphocytes, plasma cells, and eosinophils infiltrate the dense sclerotic tissue (Figure 5). This type is rare; we have encountered only 2 cases, and no other cases have been reported thus far [1, 13].

 These histological findings are somewhat similar to those characteristic of nodal IPT. Nodal IPT has been histologically classified into 3 stages (i.e., Stage I, II, and III) [14, 15]. IPT-like IgG4-related lymphadenopathy is similar to lymphadenopathy in patients with stage III nodal IPT [1, 13]. However, IPT-like IgG4-related lymphadenopathy and nodal IPT are clinically different, because patients with nodal IPT usually show symptoms that are suggestive of lymphoid malignancy (e.g., fever, fatigue, weight loss, and night sweats) [14, 15]. In contrast, patients with IPT-like IgG4-related lymphadenopathy show no symptoms suggestive of lymphoid malignancy [13]. Moreover, nodal IPT is positive for smooth muscle actin [14, 15], which further differentiates it from IPT-like IgG4-related lymphadenopathy [13].

## 3. Differential Diagnosis between IgG4-RD and Hyper-Interleukin (IL-) 6 Syndromes

**Figure 6:**
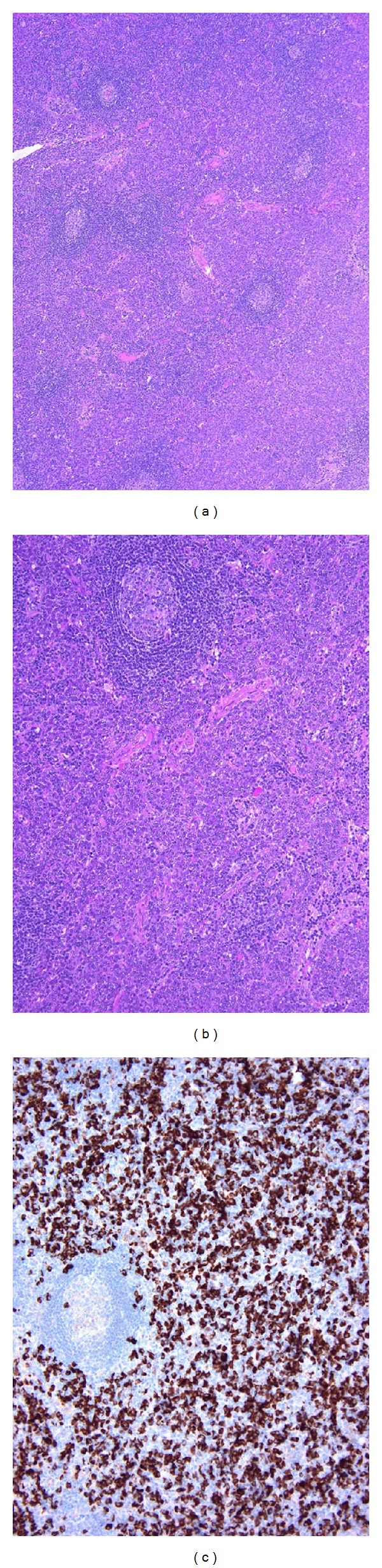
Multicentric Castleman's disease with abundant IgG4^+^ cells. (a) Atrophic germinal centers and interfollicular expansion are seen. (b) Sheets of proliferating mature plasma cells are present in the interfollicular zone. (c) The majority of mature plasma cells are positive for IgG4 (IgG4^+^/IgG^+^ plasma cell ratio >70%).

**Figure 7:**
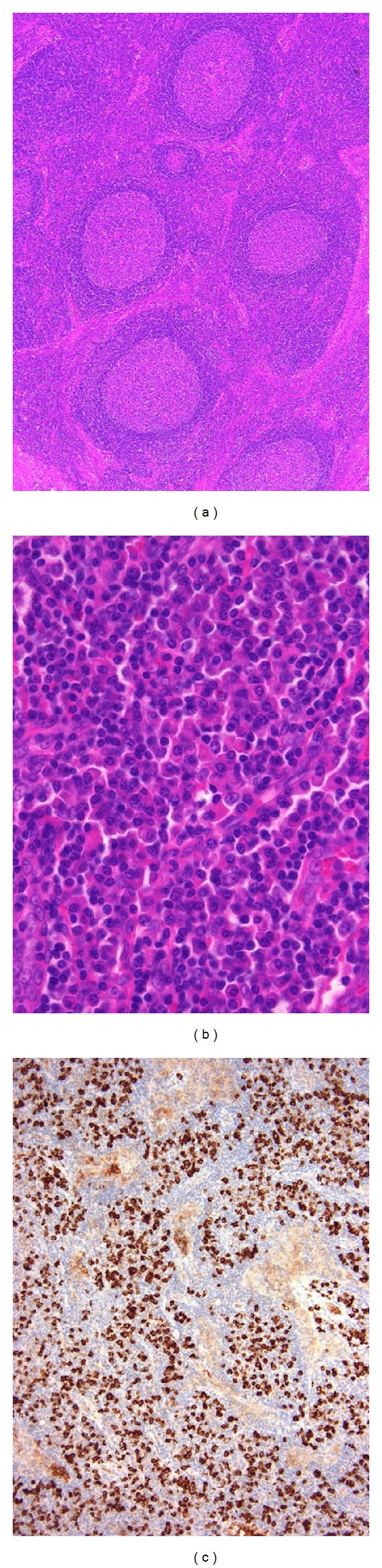
Rheumatic lymphadenopathy with abundant IgG4^+^ cells. (a), (b) The lymph node shows marked follicular hyperplasia and interfollicular plasmacytosis with small lymphocytes; eosinophil infiltration is absent. (c) The majority of mature plasma cells are positive for IgG4 (IgG4^+^/IgG^+^ plasma cell ratio >60%).

 Hyper-IL-6 syndromes such as MCD, RA, and other immune-mediated conditions are characterized by elevated serum IL-6 levels [16, 17]. Moreover, IL-6 itself functions to raise the serum levels of IgG4 and other IgG subclasses [18, 19]. In fact, MCD, RA, and other immune-mediated conditions sometimes fulfill the histological diagnostic criteria for IgG4-RD (Figures 6 and 7) and are characterized by elevated serum IgG4 levels [8, 20–23]. This complicates diagnosis, owing to the fact that hyper-IL-6 syndromes frequently involve lymph nodes. Because of this, laboratory analyses are crucial to differentiate between the 2 diseases [8]. Unlike IgG4-RD, hyper-IL-6 syndromes are characterized by elevated serum levels of IgG, IgA, IgM, and C-reactive protein (CRP); thrombocytosis; anemia; hypoalbuminemia; hypocholesterolemia (Table 2). These abnormalities are closely related to high IL-6 levels [8, 17, 20]. On the other hand, elevated serum IgE is often typical of IgG4-RD [1, 3, 5]. However, IL-6 plays a critical role in IL-4-driven IgE synthesis [24]. As such, hyper-IL-6 syndromes may also be characterized by elevated serum IgE levels, rendering serum IgE level less useful as a biomarker for a differential diagnosis of the 2 diseases [8, 22].

## 4. Conclusion

Unlike IgG4-RD that involves other organs, IgG4-related lymphadenopathy shows histological diversity, with 5 distinct subtypes. Moreover, recently, Takahashi et al. reported a unique case of IgG4-related lymphadenopathy with epithelioid granuloma [25]. This histological diversity complicates the diagnosis of IgG4-related lymphadenopathy, especially considering the similarities of the different histological subtypes to the histological characteristics of other organs involved in IgG4-RD.

Indeed, hyper-IL-6 syndromes can often fulfill the diagnostic criteria for IgG4-RD. Therefore, IgG4-RD, and especially IgG4-related lymphadenopathy, cannot be differentiated on the basis of histological findings alone. The diagnosis of IgG4-RD needs to be based not only on pathological findings but also on clinical and laboratory findings.

## Figures and Tables

**Table 1:** Histological subtypes of IgG4-related lymphadenopathy.

	Histological type	Distribution of IgG4^+^ plasma cells	Lymphadenopathy
I	Multicentric Castleman's disease-like	Interfollicular	Systemic
II	Reactive follicular hyperplasia-like	Interfollicular	Localized
III	Interfollicular expansion and immunoblastosis	Interfollicular	Systemic
IV	PTGC-type	Intragerminal center	Localized/systemic
V	Inflammatory pseudotumor (IPT-) like	Interfollicular	Localized

PTGC; progressively transformed germinal centers.

**Table 2:** Distinction between IgG4-related disease and hyper-IL-6 syndromes.

	IgG4-related disease	Hyper-IL-6 syndromes
Serum immunoglobulin	IgG↑(IgG4↑), IgE↑	IgG↑(IgG1*∼*IgG4↑), IgA↑, IgM -/↑, IgE↑
Serum IgG4/IgG ratio	Elevated	Normal (~sightly elevated)
Serum IL-6	Normal (~sightly elevated)	Elevated
Serum CRP	Normal (~sightly elevated)	Elevated
Thrombocytosis	No	Yes
Anemia	No	Yes
Hypoalbuminemia	No	Yes
Hypocholesterolemia	No	Yes

Hyper IL-6 syndromes; multicentric Castleman's disease, rheumatoid arthritis, and other immune-mediated conditions.
